# Validation of the Comprehensive Augmented Reality Testing Platform to Quantify Parkinson’s Disease Fine Motor Performance

**DOI:** 10.3390/jcm14113966

**Published:** 2025-06-04

**Authors:** Andrew Bazyk, Ryan D. Kaya, Colin Waltz, Eric Zimmerman, Joshua D. Johnston, Kathryn Scelina, Benjamin L. Walter, Junaid Siddiqui, Anson B. Rosenfeldt, Mandy Miller Koop, Jay L. Alberts

**Affiliations:** 1 Center for Neurological Restoration, Neurological Institute, Cleveland Clinic, 9500 Euclid Ave., Cleveland, OH 44195, USA; bazyka2@ccf.org (A.B.); kayar@ccf.org (R.D.K.); zimmere3@ccf.org (E.Z.); scelink@ccf.org (K.S.); walterb7@ccf.org (B.L.W.); siddiqj@ccf.org (J.S.); 2 Department of Biomedical Engineering, Lerner Research Institute, Cleveland Clinic, 9500 Euclid Ave., Cleveland, OH 44195, USA; waltzc@ccf.org (C.W.); johnstj@ccf.org (J.D.J.); rosenfa2@ccf.org (A.B.R.); koopm@ccf.org (M.M.K.)

**Keywords:** Parkinson’s disease, motor assessment, finger tapping, augmented reality, motion capture

## Abstract

**Background/Objectives**: Technological approaches for the objective, quantitative assessment of motor functions have the potential to improve the medical management of people with Parkinson’s disease (PwPD), offering more precise, data-driven insights to enhance diagnosis, monitoring, and treatment. Markerless motion capture (MMC) is a promising approach for the integration of biomechanical analysis into clinical practice. The aims of this project were to evaluate a commercially available MMC system, develop and validate a custom MMC data processing algorithm, and evaluate the effectiveness of the algorithm in discriminating fine motor performance between PwPD and healthy controls (HCs). **Methods**: A total of 58 PwPD and 25 HCs completed finger-tapping assessments, administered and recorded by a self-worn augmented reality headset. Fine motor performance was evaluated using the headset’s built-in hand tracking software (Native-MMC) and a custom algorithm (CART-MMC). Outcomes from each were compared against a gold-standard motion capture system (Traditional-MC) to determine the equivalence. Known-group validity was evaluated using CART-MMC. **Results**: A total of 82 trials were analyzed for equivalence against the Traditional-MC, and 152 trials were analyzed for known-group validity. The CART-MMC outcomes were statistically equivalent to Traditional-MC (within 5%) for tap count, frequency, amplitude, and opening velocity metrics. The Native-MMC did not meet equivalence with the Traditional-MC, deviating by an average of 24% across all outcomes. The CART-MMC captured significant differences between PwPD and HCs for tapping amplitude, amplitude variability, frequency variability, finger opening and closing velocities, and their respective variabilities, and normalized path length. **Conclusions**: The biomechanical data gathered using a commercially available augmented reality device and analyzed via a custom algorithm accurately characterize fine motor performance in PwPD.

## 1. Introduction

Parkinson’s disease (PD) is a neurodegenerative disease characterized by tremor, postural instability and gait difficulties (PIGD), rigidity and bradykinesia. Bradykinesia is a particularly challenging symptom to precisely characterize in clinical practice [1] as it manifests in multiple motor impairments, including hypokinesia, slowness of movement, and arrhythmicity [2]. Impairments in coordinated, rhythmic fine motor movements impact performance of activities of daily living (ADLs) and instrumental ADLs (IADLs) in people with PD (PwPD) [3]. Thus, bradykinesia negatively impacts overall quality of life [4]. Additionally, imaging studies indicate finger tapping rhythmicity corresponds to the integrity of dopaminergic neurons in the basal ganglia [5]. Considering that finger-tapping performance provides insight into both daily functions and basal ganglia circuity, it is an important marker of PD severity, progression, and treatment efficacy.

The current gold-standard clinical assessment of finger tapping is item 3.4 of the Movement Disorders Society-Unified Parkinson’s Disease Rating Scale Part III (MDS-UPDRS III) [6]. Scoring requires a clinician to detect and rate the subtleties of movements that are ill-suited for real-time visual quantification (e.g., speed, amplitude, hesitations, and amplitude decrements). Following visual monitoring, the clinician assigns an ordinal score between 0 (no impairment) and 4 (severe impairment). Despite its broad clinical acceptance, the objectivity [7], sensitivity [8], and reliability [9] of the MDS-UPDRS III scoring have been questioned.

Sensor-driven products aimed to objectively assess fine motor functions have been developed to address the limitations of the MDS-UPDRS III [10]. However, most technologies have failed to be integrated into clinical practice [11]. Although they provide objective and quantifiable outcomes of motor performance, technological approaches generally lack validation against a gold-standard reference system [12]. Forty years have passed since Ward et al. proposed several fundamental points regarding the development of technologies for PD assessment that remain relevant but largely unmet: PD-related technologies should evaluate important patient-centered performance metrics, provide information that cannot otherwise be obtained through current clinical methods, be sensitive to subtle changes in symptom presentation, and have promise for clinical integration [13]. Despite this call for improved assessment methods, a scoping review found that less than 6% of technological approaches demonstrated the potential for clinical integration [14]. Thus, while accurate, an objective quantification of motor symptoms is critical for informing and advancing clinical decision-making for PwPD, it remains an unrealized goal in clinical practice.

Technological advancements in camera hardware and human pose estimation software have facilitated the use of markerless motion capture (MMC) for biomechanical analyses [15]. Markerless motion capture systems have the potential to standardize and democratize the use of biomechanical outcomes to quantify PD motor functions in clinical settings. Markerless motion capture leverages red–green–blue (RGB) cameras, depth cameras, or a combination of RGB and depth cameras to collect biomechanical data. Three-dimensional position data, reconstructed from camera images and parameters, are then processed with pose estimation software, such as MediaPipe [16], OpenPose [17], or custom post-processing pipelines [18]. Compellingly, MMC systems can record positional data for biomechanical analysis with relatively inexpensive commercially available devices, like smartphones, tablets, or augmented reality (AR) headsets. The Microsoft HoloLens 2 (HL2) AR headset contains a native MMC hand tracking algorithm (Native-MMC); however, this software lacks accuracy when compared to traditional 3D motion capture systems [19]. Notably, the Native-MMC hand tracking algorithm was developed to detect basic hand gestures (e.g., pointing, grasping, and pinching) for interfacing with the device, not for capturing precise biomechanical data to quantify motor performance. Thus, continuous hand and finger movements used to evaluate fine motor functions in PwPD push the limitations of the Native-MMC hand tracking, underscoring the need to develop custom data processing algorithms for research and clinical applications.

To analyze finger-tapping data, a custom MMC algorithm (CART-MMC) was developed to overcome the limitations of the Native-MMC hand tracking. Briefly, the Comprehensive Augmented Reality Testing (CART) platform consists of multiple assessment modules to elicit and quantify upper and lower extremity PD motor symptoms, including tremor, bradykinesia, and PIGD. This manuscript focuses on data from the CART finger-tapping module to evaluate bradykinesia. The aims of the manuscript are (1) to evaluate the Native-MMC in the quantification of fine motor movements, (2) to detail the methodology for collecting and analyzing the CART-MMC data, (3) to assess the accuracy of the CART-MMC compared to a gold-standard motion capture system, and (4) to determine the known-group validity of the CART-MMC algorithm to distinguish PwPD from healthy controls (HCs).

## 2. Methods

### 2.1. Participants

A total of 83 participants completed finger-tapping assessments (25 HCs, 58 PD). Seventy-nine were included for analysis (Table 1). Inclusion criteria for the PD group included (1) diagnosis of idiopathic PD, (2) Hoehn and Yahr stage I–IV [20], (3) absence of other neurological conditions, (4) ability to follow 2-step commands, and (5) stable antiparkinsonian medication regimen for a minimum of one month prior to assessment. Inclusion criteria for the HC group included (1) absence of any neurological condition and (2) ability to follow 2-step commands. For both groups, exclusion criteria included (1) implanted deep brain stimulation device, (2) history of a gait-altering musculoskeletal injury, or (3) uncorrected vision or hearing impairments that would impact interaction with an AR headset. This study was approved by the Cleveland Clinic IRB (protocol code 23-1146, date of approval: 8 December 2023)., and all participants completed the informed consent process prior to data collection.

The PD group was tested in the off-medication state: antiparkinsonian medications withheld 12 h prior to data collection. Parkinson’s disease motor symptom severity was assessed with the MDS-UPDRS III by an MDS-certified examiner. Disease severity was classified according to the criteria established by Martínez-Martín et al. (mild = MDS-UPDRS III score of 0–32, moderate = 33–58, and severe = 59+) [21].

### 2.2. Finger-Tapping Assessment Set-Up and Procedure

**Figure 1:**
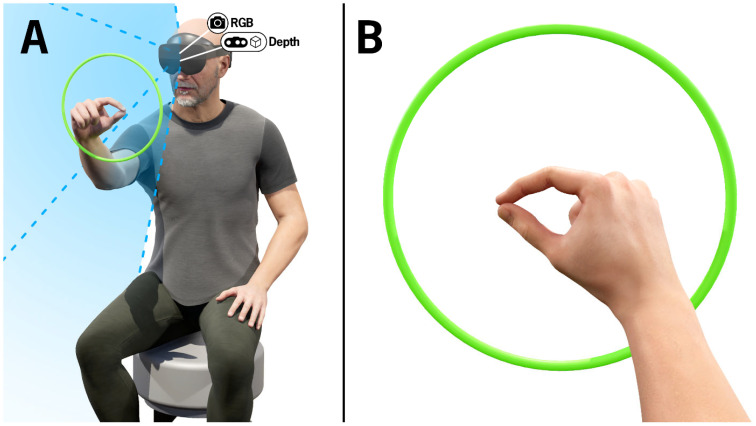
(**A**) Visualization of a participant completing the finger-tapping test. The green ring represents recognition of the hand in view of the HL2 RGB and depth cameras and is only visible to the wearer of the HL2. The blue shaded region depicts the cameras’ field of view. The camera icons indicate the position of the RGB and depth cameras embedded in the HL2. (**B**) Visualization of the finger-tapping test from the perspective of the participant wearing the HL2.

All participants completed two finger-tapping trials, one with each hand, while wearing the HL2 (Microsoft, Redmond, WA, USA) (Figure 1). To ensure uniformity, all task instructions were delivered via HL2-embedded speakers while an avatar hand demonstrated the finger-tapping task. As with the MDS-UPDRS III finger-tapping assessment, participants were instructed to repetitively tap their thumb and index finger together as big and as fast as possible. The participant was instructed to position their hand in a ring, displayed in the AR headset, to ensure the tapping was in the field of view of the headset cameras. The color of the ring changed from red to green when the hand was positioned correctly.

Following a 10 s practice trial, right- and left-hand trials were completed, each 20 s in duration. The supervising clinician also wore an AR headset and, following each trial, was provided a percentage of the trial that the participant’s hand was in view. If the percentage of time within view of the camera was less than 75%, the trial was repeated. If needed, the participant was provided a table for upper-extremity stability to minimize extraneous movement (i.e., tremor or dyskinesias).

Cameras and sensors embedded in the HL2 recorded hand landmark positions using Microsoft’s Native-MMC hand tracking as well as the RGB images, depth images, and camera parameters used for the CART-MMC. Six retro-reflective markers were placed on the dorsum of each hand: 1st digit distal phalanx (thumb tip), 2nd digit distal phalanx (index fingertip), 2nd intermetacarpal space, 4th intermetacarpal space, radial styloid, and ulnar styloid. The thumb tip and index fingertip markers were used for the Traditional-MC outcome calculations, and the remaining four markers provided spatial anchors used for post-processing the data. The traditional-MC utilized sixteen motion capture cameras (Vicon Motion Systems, Oxford, UK) to record the retro-reflective marker positions at 100 Hz. Data streams from the HL2 and Traditional-MC system were synchronized via trial initiation and termination triggers sent over a local network.

### 2.3. CART Platform Development

The CART HL2 application was developed in Unity 2021.3.4f1 using C#, Microsoft Mixed Reality Toolkit 3.0.0-pre14, Microsoft Mixed Reality OpenXR Plugin 1.8.0, and Unity OpenXR Plugin 1.6.0. All software was developed within Windows Holographic operating system version 23H2, build number 22621.1258.

### 2.4. Native-MMC Data Collection

Hand position data were collected at 60 Hz using the Microsoft Mixed Reality Toolkit OpenXR Hands Subsystem class. The subsystem retrieved positional data for 25 landmarks on each hand, including the index fingertip and thumb tip, in real-time from the Microsoft Mixed Reality OpenXR plugin, an extension of the Unity OpenXR plugin. This plugin received information from the OpenXR runtime to provide access to the HL2 device capabilities. The Native-MMC 3D hand landmark positions were saved to a CSV file after each trial was completed.

### 2.5. CART-MMC Data Collection and 3D Hand Landmark Identification

RGB images, depth images, and camera parameters were collected from the HL2 using Research Mode and processed offline in MATLAB to reconstruct the CART-MMC hand model.

#### 2.5.1. Data Collection of Images and Camera Parameters

Research Mode was used to access the HL2 camera images and parameters necessary for the CART-MMC analysis. A dynamic-link library plugin written in C++ used the Research Mode API to obtain the camera related data. Red–green–blue images were collected at 30 Hz from the HL2 RGB camera with a resolution of 640 × 360. To acquire depth data, Articulated Hand Tracking (AHaT) images, in near-depth sending mode, were sampled at 45 Hz with a resolution of 512 × 512. Red–green–blue images were saved as binary data in bytes files, and depth images were saved as Portable Gray Map (PGM) files. Two tar files were created to store these RGB and depth data, respectively. In addition to the RGB and depth images, RGB and depth camera parameters were saved to text files.

The following RGB camera parameters were saved: Cam2World_RGB_, Timestamps_RGB_ and K_RGB_. Cam2World_RGB_ contained the transformation from RGB camera coordinates to world coordinates for each RGB image. Timestamps_RGB_ defined the time at which each RGB image was captured. K_RGB_ contained the intrinsics necessary for conversion of RGB camera coordinates to RGB image coordinates.

The following depth camera parameters were output from the HL2: Look-Up-Table (LUT), Rig2Cam_Depth_, Rig2World_Depth_, and Timestamps_Depth_. The Research Mode API did not provide the intrinsic parameters of the depth camera directly. Instead, the LUT contained the 3D camera unit coordinates for each pixel in the depth image, estimated using a Levenberg–Marquardt optimization. The Rig2Cam_Depth_ transformation was used to convert between depth camera and rig coordinates. Timestamps_Depth_ contained the timestamp for each depth image. Rig2World_Depth_ contained a transformation from the rig to the world coordinate system for each depth image captured.

#### 2.5.2. Image Extraction and Temporal Alignment

**Figure 2:**
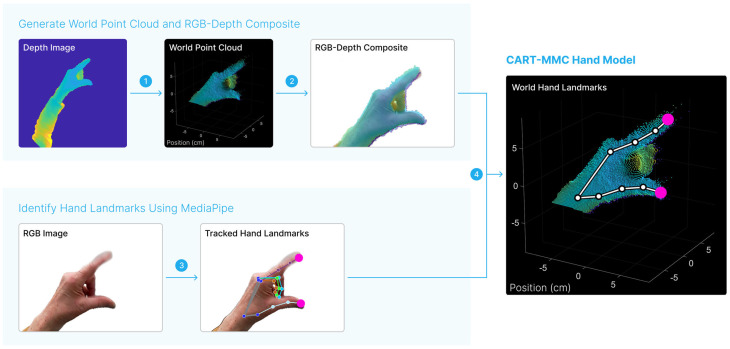
Representative temporally aligned depth and RGB images are shown. First, in step 1, the depth image was converted to a 3D point cloud in the world space. In step 2, the world point cloud positions were projected onto the RGB image, creating a composite RGB-depth image. MediaPipe was used to identify hand landmark positions in step 3 from the RGB image. The landmark positions of interest, the index fingertip and thumb tip are indicated in pink. The MediaPipe-identified landmark positions were mapped to the world space using the RGB-depth composite in step 4, yielding the CART-MMC hand model composed of 21 key landmark 3D positions in world space. The process was repeated for each RGB image.

The CART-MMC analysis was performed offline in MATLAB (MathWorks, MATLAB R2022b). Figure 2 illustrates the processing steps performed to achieve the 3D CART-MMC hand model. First, RGB images were extracted from the tar file into individual bytes image files then converted to PNG images. Depth images were extracted from the tar file and converted from PGM images to PNG images. For each RGB image, the closest temporal depth image was matched by the minimum absolute difference between the current RGB timestamp and Timestamps_Depth_. Construction of the 3D hand was performed for each RGB and depth image pair.

#### 2.5.3. Convert Depth Image to World Point Cloud

Depth image points were converted to points in the depth camera unit space using the LUT (Equation (1)). For each image point, the LUT contained the corresponding camera unit space points. Depth camera unit space points were multiplied (elementwise matrix multiplication) by the corresponding depth value to obtain depth camera points, for which the length of each projected ray was equal to the depth value (Equation (2)). The Rig2Cam_Depth_ and Rig2World_Depth_ transformations were used to transform depth camera points to world points (Equation (3)). Step 1 in Figure 2 shows a representative world point cloud generated from a depth image using Equations (1)–(3).

(1) $${\left[\begin{array}{c} \vec{u}\\ \vec{v}\\ \vec{1} \end{array}\right]}_{Depth\_Image}\overset{LUT}{\to }{\left[\begin{array}{c} \vec{x}\\ \vec{y}\\ \vec{z} \end{array}\right]}_{Depth\;\_CamUnit}$$

(2) $${{\left[\begin{array}{c} \vec{x}\\ \vec{y}\\ \vec{z} \end{array}\right]}_{Depth\_Cam}=\left[\begin{array}{c} \vec{x}\\ \vec{y}\\ \vec{z} \end{array}\right]}_{Depth\_CamUnit}\circ \left[\begin{array}{c} \vec{d}\\ \vec{d}\\ \vec{d} \end{array}\right]$$

(3) $${{\left[\begin{array}{c} \vec{x}\\ \vec{y}\\ \vec{z}\\ \vec{1} \end{array}\right]}_{World}=\left({Rig2World}_{Depth}\right)\times {\left({Rig2Cam}_{Depth}\right)}^{-1}\times \left[\begin{array}{c} \vec{x}\\ \vec{y}\\ \vec{z}\\ \vec{1} \end{array}\right]}_{Depth\_Cam}$$

#### 2.5.4. Calculate RGB-Depth Composite

World points were then projected to the RGB camera point space using the inverse of the Cam2World_RGB_ transformation (Equation (4)). The K_RGB_ matrix was used to obtain RGB image points (Equation (5)), generating the RGB-depth composite image.

(4) $${\left[\begin{array}{c} \vec{x}\\ \vec{y}\\ \vec{z}\\ \vec{1} \end{array}\right]}_{RGB\_Cam}={{\left({Cam2World}_{RGB}\right)}^{-1}\times \left[\begin{array}{c} \vec{x}\\ \vec{y}\\ \vec{z}\\ \vec{1} \end{array}\right]}_{World}$$

(5) $${\left[\begin{array}{c} \vec{u}\\ \vec{v}\\ \vec{z} \end{array}\right]}_{RGB\_Image}={K_{RGB}\times \left[\begin{array}{c} \vec{x}\\ \vec{y}\\ \vec{z} \end{array}\right]}_{RGB\_Cam}$$

Points deprojected from the depth image and reprojected on the RGB image that fell outside the RGB image size were considered invalid and removed. The remaining points comprised the RGB-depth composite, shown in Figure 2.

#### 2.5.5. Identify Hand Landmark Positions in the RGB Image with MediaPipe

The RGB images were tracked using Google MediaPipe hand landmark estimation software (Version 0.10.13). The software identified 21 hand landmark positions, including the index fingertip and thumb tip, for each RGB image. The positions were saved as normalized units (0, 1) and converted to the RGB image coordinate space using the RGB image width and height. The “Tracked Hands Landmarks” image in Figure 2 shows the 21 hand landmarks identified by MediaPipe.

#### 2.5.6. 3D World Hand Landmark Positions

For the MediaPipe hand landmark positions, the nearest projected RGB image points in the RGB-depth composite were identified. The corresponding world points defined the CART-MMC hand model (“World Hand Landmarks”, Figure 2).

#### 2.5.7. Interpolation

Given the depth camera’s sample rate of 45 Hz, the theoretical maximum temporal offset between an RGB image capture and a depth image capture was 11.1 ms, and RGB-depth image pairs separated by more than 11.1 ms indicated a missing depth image. Additionally, depth values outside the range of 15–45 cm were considered invalid. In both cases, 3D positions for those samples were linearly interpolated.

### 2.6. Traditional-MC Data Processing

Following data collection, the Traditional-MC data was processed with Vicon software. The raw marker positions were reconstructed, and the six markers were labeled for each trial. This data was then manually reviewed to ensure data integrity (e.g., complete, correctly labeled tracking) prior to exporting.

### 2.7. Metric Calculations

The data analysis process was consistent across the three motion capture systems. Position data for all hand landmarks were first resampled to 60 Hz using linear interpolation. The first 2 s and last 1 s of each trial were removed. The 3D distance between thumb tip and index fingertip (**Distance-Thumb-To-Index**) was calculated using the Euclidean distance formula. Data were centered on the mean for temporal alignment. Temporal alignment was achieved by shifting the data to maximize the cross-correlation between **Distance-Thumb-To-Index**, calculated using the *xcorr* MATLAB function. The signals were cut to the minimum length between the three systems (excess data at front and end were removed). **Distance-Thumb-To-Index** was filtered using a 4th order Butterworth low pass filter with a cutoff frequency of 5 Hz. A measure of velocity was estimated by computing the central difference approximation on the **Distance-Thumb-To-Index**.

**Figure 3:**
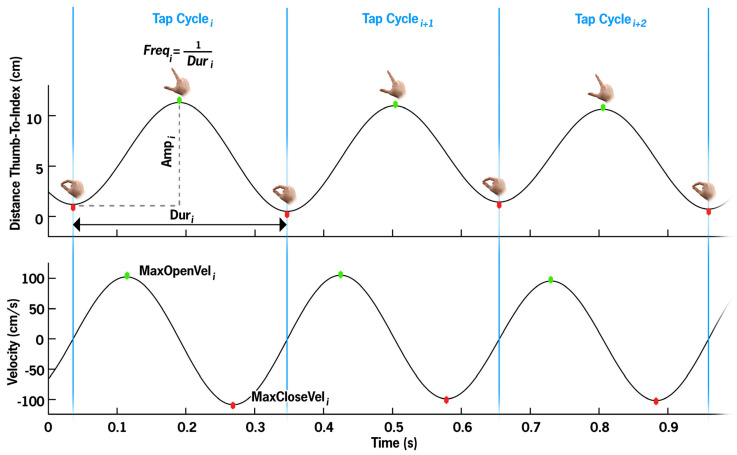
Per cycle metrics visualized. The top panel illustrates the distance between the thumb and index finger, with each apex representing the maximum thumb–index finger separation (green dot) per tapping cycle, and the trough representing the closure of the thumb and index finger (red dot). The bottom panel depicts the movement velocity, with the maximum velocity generally occurring near the midpoint of the opening segment, and the maximum closing velocity occurring near the midpoint of the closing segment. One TapCycle, segmented by blue bars, is defined as finger close to finger close (local minima in Distance-Thumb-To-Index). Dur_i_ is the time between cycle start and stop. Freq is the inversion of Dur. Amp is defined as the maximum distance minus the initial closed distance. MaxOpenVel and MaxCloseVel are the local maximum and minima velocity values, respectively.

Kinematic metrics were calculated for the left and right hands separately. Local minima detection was performed using the *findpeaks* MATLAB function on the **Distance-Thumb-To-Index** with a minimum peak prominence of 0.5 cm, and a minimum peak separation of 0.15 s. Two consecutive minima defined one **TapCycle**. For each **TapCycle**, duration (**Dur**), frequency (**Freq**), and amplitude (**Amp**) were calculated. **Amp** (cm) was defined as the difference between the max open position and the initial close position. **Dur** (s) is the temporal difference between the final close position and the initial close position. **Freq** (Hz) is the inverse of **Dur**. **MaxOpenVel** and **MaxCloseVel** are the local maximum and minimum velocities per cycle, respectively (Figure 3).

**TapCount** is the total number of tap cycles completed. Mean and coefficients of variation (CV) were calculated for **Amp**, **Freq**, **MaxOpenVel**, and **MaxCloseVel** (**Amp-Mean**, **Amp-CV**, **Freq-Mean**, **Freq-CV**, **MaxOpenVel-Mean**, **MaxOpenVel-CV**, **MaxCloseVel-Mean**, **MaxCloseVel-CV**). Normalized path length (**NPL**) is the sum of the absolute value of the 3D difference between consecutive distances divided by total trial duration, characterizing both movement amplitude and velocity.

### 2.8. Statistical Analysis

The outcomes from the Native-MMC and CART-MMC systems were compared against the Traditional-MC to determine the accuracy of each MMC system. Following the equivalence validation, differences between the HC and PD groups were analyzed using the CART-MMC outcomes. Overall descriptive summaries were computed for each metric with individual participant data collapsed across hands, then reported as means and standard deviations. Analysis was conducted using RStudio 2024.12.1, R version 4.4.3.

#### 2.8.1. System Equivalence Statistical Analysis

To determine the accuracy of the Native-MMC and CART-MMC systems against the Traditional-MC, outcomes were tested for equivalence between systems using the bounds of ±5% of the Traditional-MC. For each MMC system and outcome, equivalence was tested using a linear mixed model with fixed terms of system and group and random intercepts by participant and trial for each participant. Denominator degrees of freedom were estimated using the Kenward–Roger approximation.

#### 2.8.2. Known-Group Statistical Analysis

Group differences between HCs and PwPD were assessed with a linear mixed model for each outcome using the CART-MMC data. The linear mixed models included a fixed effect for group and random intercepts per participant. Denominator degrees of freedom were estimated using the Kenward–Roger approximation.

### 2.9. Final Dataset for Analyses

Of the 166 trials collected from all 83 participants, 14 (8%) of the trials were removed (3 HCs, 11 PD) due to technical errors (e.g., participant’s hand outside of the camera field of view, files not saved, failure of MMC to identify hand), resulting in 152 trials from 79 participants (24 HCs, 55 PD) included in the known-group analysis. An equivalence analysis was conducted on a subgroup of participants (20 HCs, 24 PD), comparing the Traditional-MC outcomes to the Native-MMC and CART-MMC outcomes, respectively.

## 3. Results

### 3.1. Native-MMC Outcomes Lack Equivalence with the Traditional-MC

All finger-tapping outcomes from the Native-MMC deviated from the Traditional-MC by more than 5% (all *p* > 0.50) (Table 2). The Native-MMC measured a 7.6% lower **TapCount** than the Traditional-MC, a 5.4% lower **Freq-Mean**, and a 20% smaller **Amp-Mean**. **MaxOpenVel-Mean**, **MaxCloseVel-Mean,** and **NPL** were underestimated by 23%, 24%, and 23%, respectively. Variability was overestimated by at least 28%, evidenced by the CV outcomes.

### 3.2. CART-MMC Outcomes Align Closely with the Traditional-MC

**Figure 4:**
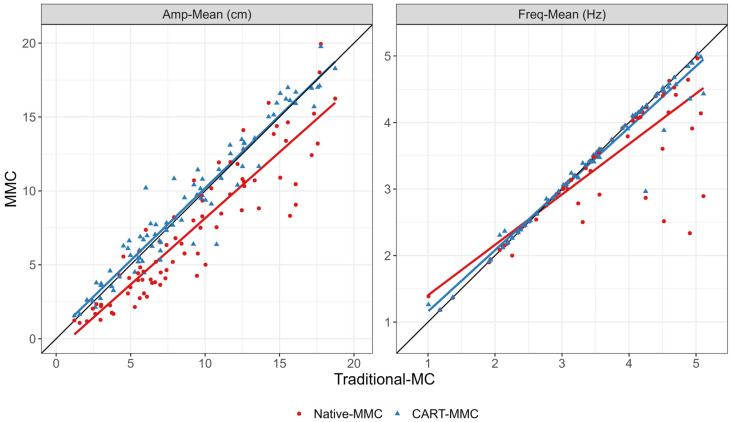
Scatterplots and linear fit lines for **Amp-Mean** and **Freq-Mean** for each trial, comparing the Traditional-MC against the Native-MMC (red dots and red line) and the CART-MMC (blue triangles and blue line). The black reference lines represent perfect equivalence with the Traditional-MC. **Left plot:** The Native-MMC consistently underestimated **Amp-Mean**, by 19.5% on average, depicted by the red line below the equivalence line. The CART-MMC measured **Amp-Mean** within 1.7% on average, indicated by the blue line’s close alignment to the equivalence line. **Right plot:** The Native-MMC’s **Freq-Mean** showed poor accuracy above 3 Hz, represented by the red line skewing away from the equivalence line. The CART-MMC accurately quantified **Freq-Mean** across the range of frequencies measured, demonstrated by the close alignment of the blue line with the equivalence line.

For **TapCount**, **Amp-Mean**, **Freq-Mean**, **MaxOpenVel-Mean**, **MaxCloseVel-Mean**, and **NPL**, the mean difference between systems was within the 5% equivalence range. **TapCount**, **Amp-Mean**, **Freq-Mean**, and **MaxOpenVel-Mean** were significantly equivalent (*p* < 0.05), within the 5% range. The CART-MMC **NPL** outcome was within 2.4% of the Traditional-MC (equivalence *p* = 0.06). For **MaxCloseVel-Mean**, the measured difference was 3.1% greater in the CART-MMC (equivalence *p* = 0.15). For the CV outcomes (**Amp-CV**, **Freq-CV**, **MaxOpenVel-CV**, and **MaxCloseVel-CV**), the percent difference between systems was 17%, 29%, 20%, and 19% larger than the Traditional-MC, respectively (all equivalence *p* > 0.99). Figure 4 provides all trial data plotted against the Traditional-MC for **Amp-Mean** and **Freq-Mean**.

### 3.3. CART-MMC Differentiates Finger-Tapping Performance in PwPD from HCs

**Figure 5:**
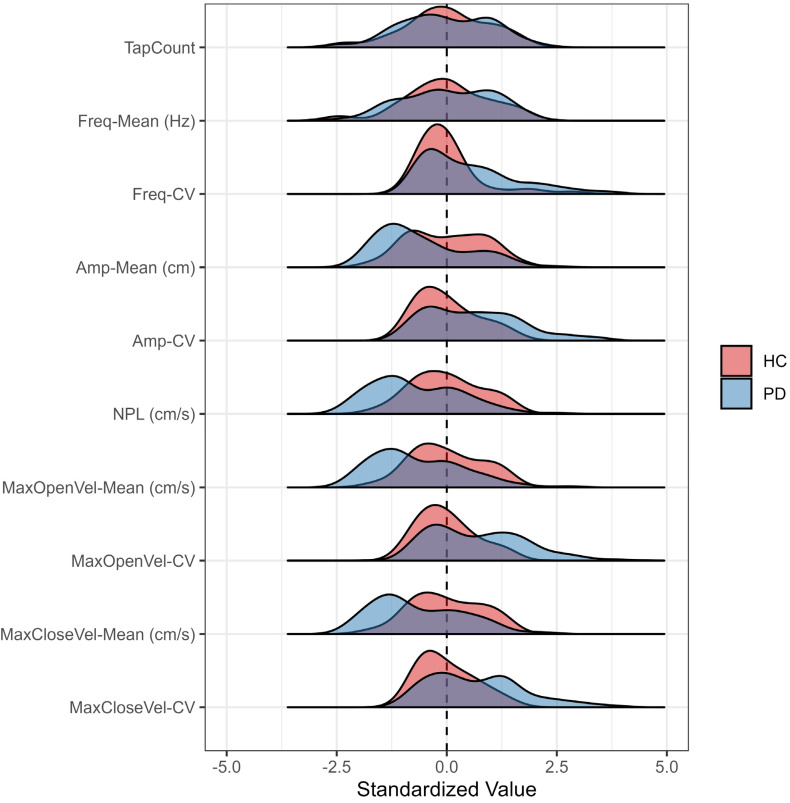
Outcome distributions measured by the CART-MMC for HCs and PwPD, presented as the count of pooled standard deviations away from the HC mean set at zero. Compared to HCs, PwPD demonstrated a similar distribution for **Tap-Count** and **Freq-Mean**. PwPD demonstrated slower, reduced movement, evidenced by left-skewed **Amp-Mean**, **NPL**, **MaxOpenVel-Mean**, and **MaxCloseVel-Mean** distributions. PwPD demonstrated more variable (i.e., dysrhythmic) movements with **Freq-CV**, **Amp-CV**, **MaxOpenVel-CV**, and **MaxCloseVel-CV** distributions extending to four pooled standard deviations away from the HC mean.

Measured by the CART-MMC, HC and PD **TapCount** and **Freq-Mean** per trial [mean (SD)] were comparable: 46.4 (12.2) vs. 46.3 (12.5), respectively (*p* = 0.96), and 3.38 Hz (0.84) vs. 3.42 Hz (0.87), respectively (*p* = 0.84). Participants with PD exhibited a 25% smaller **Amp-Mean**, were 44% slower in both **MaxOpenVel-Mean** and **MaxCloseVel-Mean**, had a 26% smaller **NPL**, and at least 36% more variability, evidenced by the CV outcomes (all *p* < 0.05) (Table 3). Figure 5 demonstrates that CART-MMC is sensitive to hypokinesia, slowness of movement, dysrhythmia, and increased variability in PwPD.

## 4. Discussion

The CART-MMC leverages RGB and depth camera data from an AR headset to provide an accurate biomechanical analysis of finger-tapping performance in PwPD. Valid biomechanical data from a single device can facilitate the acquisition of quantitative, objective outcomes both within and beyond traditional clinical settings to characterize and monitor PD fine motor function and bradykinesia.

Consistent with the previous findings [19], the HL2’s Native-MMC demonstrated poor equivalence to the Traditional-MC, with an average deviation of 24% across outcomes. The lack of equivalence suggests the Native-MMC is unable to capture the important movement qualities associated with PD. Bradykinetic, high frequency, and dysrhythmic movements associated with PD differ from the gross motor hand gestures used to operate the HL2. Quantification of fine upper extremity movements, aimed at informing clinical decision-making, requires the appropriate coupling of data collection and processing technology with the types of movement being evaluated. Thus, commercial technology should be examined for validity across specific movements that are of clinical interest. Without proper validation, measurement system limitations remain unknown, and can result in inaccurate outcomes and misguided interpretation of those outcomes. If technology is to be used to aid clinical decision-making, the data from that technology must be validated and its outcomes trusted.

The biomechanical outcomes of tap count and the means of amplitude, frequency, maximum opening and closing velocities, and NPL measured via CART-MMC were within 5% of the gold-standard Traditional-MC system. Clinical adoption of MMC systems relies on rigorous validation of important and relevant outcomes. Previous studies have relied on correlating MMC outcomes with MDS-UPDRS III scores [22,23]. While the MDS-UPDRS III is the clinical gold standard for rating PD symptoms, it falls short in accurate movement quantification. The gold standard in quantifying movement is traditional 3D motion capture. Previous reports have validated MMC against gold-standard motion capture systems for the finger-tapping test [24]. However, these MMC systems employed 2D data analysis, which provides accurate temporal data but cannot measure depth-dependent metrics, such as tap amplitude, opening and closing velocities, and NPL. By contrast, by integrating the depth camera data, the CART-MMC accurately quantifies depth-dependent measures. Similarly, in a cohort of young, healthy adults, Amprimo et al. found a strong correlation of finger-tapping metrics between the depth-enhanced Google MediaPipe Hand framework and a gold-standard motion capture system [25]. Building upon the healthy younger adult analysis, outcomes from the CART finger-tapping assessment demonstrate that the depth-enhanced CART-MMC algorithm accurately assesses and quantifies unconstrained finger tapping in PwPD.

In addition to providing valid, biomechanically appropriate metrics, the CART-MMC was sensitive to PD-specific deficits, successfully discriminating between the HC and PD groups. Tap count and its derivative, tap frequency, did not differ between the groups; however, the number of taps in a period is not a particularly informative measure of symptom severity, or even of distinguishing PD [26]. In PwPD, repetitive motor tasks often result in a sequence effect, or loss of speed or amplitude with sequential movements [27]. Thus, a reliance on tap count alone ignores the aggregate of inter-dependent impairments that impact finger-tapping performance, and ultimately bradykinesia and manual function. With an expanding conception of bradykinesia from slowness of movement to a more robust definition that includes hypokinesia, sequence effect, and dysrhythmia [2], the CART-MMC provides objective, quantifiable data that captures the bradykinesia complex in PwPD. The data provided in Figure 5 provide meaningful outcomes that satisfy the hallmarks of the bradykinesia complex: hypokinesia as reflected by the lower tap amplitude and NPL relative to HCs, slowness as measured by the velocity outcomes, and sequence effect and dysrhythmia as measured by all CV outcomes. The CART-MMC accurately quantifies the nuances of the bradykinesia complex, consistent with known-group differences observed in previous studies [28,29,30].

Augmented reality headsets, such as the HL2 and others, contain the necessary hardware to leverage MMC technology to routinely gather biomechanical data to precisely characterize movement dysfunction and performance in neurological populations such as PD. The CART application was created using the Develop with Clinical Intent (DCI) medical software development model [11]. Paramount to the DCI model is that any technology developed must address a known gap in clinical care, be conducive to integration into current clinical workflows, and facilitate the collection of a set of standardized outcomes that can be used to advance the understanding or treatment of a disease.

The CART platform can be deployed in a busy movement disorders clinic by providing the self-administered finger-tapping and other assessments to the patient prior to their appointment in the area formerly known as the “waiting room”. This concept of transforming a space in which patients simply wait prior to the appointment with their provider is already taking place within the Neurological Institute at the Cleveland Clinic [11]. The CART platform and similar technologies have the capacity to improve the care of those in rural and underserved areas. Borrowing from the home sleep testing model, one can envision sending an AR headset to the home of the patient. The patient could complete the self-administered assessment modules, and those data could be immediately uploaded to a secure server, processed and integrated into the patient’s electronic health record for their provider to review and discuss during a virtual visit. A validated remote approach for assessing PD symptoms is critical as only 10% of PwPD have an annual visit with a movement disorders neurologist, and more than 50% are not seen by any neurologist [31]. Fundamentally, augmenting clinical judgment with objective and quantitative outcomes that characterize important motor, and eventually cognitive, functions has the potential to democratize high quality treatment for PD patients regardless of their disease severity or proximity to a large academic medical center. Considering the importance of this expansion of high-quality PD treatment, future studies should leverage the CART platform for evaluation across broader disease severity and geographical ranges.

The HL2 was selected for this study as the device has been validated for gait assessment in PwPD [32,33]. However, it is fully appreciated that other existing and future systems provide RGB and depth data, and the CART-MMC data processing algorithm is suitable for use in processing data from these systems. Furthermore, deficits in fine motor movements are not exclusive to PD. The CART-MMC, with proper validation, can serve to objectively evaluate motor performance and track changes in function in conditions such as multiple sclerosis, cerebral vascular accidents, and others that result in fine motor impairments.

## 5. Conclusions

The CART finger-tapping module and the CART-MMC data processing algorithm provide accurate biomechanical data to assess movement speed, amplitude, and frequency during a finger-tapping test in HCs and PwPD. The equivalence and known-group validation of this CART-MMC algorithm position it as a reliable tool for objectively quantifying PD motor symptoms and their progression to inform and broaden access to clinical care.

## Figures and Tables

**Table 1:** HC and PD group demographics.

	Healthy Controls (*n* = 24)	Parkinson’s Disease (*n* = 55)
**Age** (years)	68.6 (6.0)	68.2 (7.9)
**Gender**		
Female	16 (66.7%)	13 (23.6%)
Male	8 (33.3%)	42 (76.4%)
**Race**		
White	24 (100%)	50 (90.9%)
Black	0 (0%)	5 (9.1%)
**Ethnicity**		
Not Hispanic or Latino	24 (100%)	55 (100%)
**Education** (years)	17.0 (2.9)	17.1 (2.3)
**MDS-UPDRS III Total Score**	-	36.2 (13.6)
**Severity Score**		
Mild (0–32)	-	22 (40.0%)
Moderate (33–58)	-	29 (52.7%)
Severe (59+)	-	4 (7.3%)

Data summarized as a mean (SD) or *n* (%).

**Table 2:** CART-MMC outcomes demonstrate equivalence to Traditional-MC.

	Traditional-MC	Native-MMC			CART-MMC		
Outcome	Mean (SD)	Mean (SD)	% Difference from Traditional-MC	Equivalence *p*-Value	Mean (SD)	% Difference from Traditional-MC	Equivalence *p*-Value
TapCount	46.4 (13.5)	42.9 (12.3)	−7.6%	0.89	45.6 (12.6)	−1.8%	<0.001 **
Freq-Mean (Hz)	3.37 (0.96)	3.19 (0.83)	−5.4%	0.58	3.35 (0.89)	−0.6%	<0.001 **
Freq-CV	0.11 (0.06)	0.16 (0.12)	45.2%	>0.99	0.15 (0.08)	28.7%	>0.99
Amp-Mean (cm)	8.94 (4.49)	7.20 (4.38)	−19.5%	>0.99	9.09 (4.48)	1.7%	0.035 *
Amp-CV	0.26 (0.14)	0.33 (0.15)	28.0%	>0.99	0.30 (0.13)	16.9%	>0.99
NPL (cm/s)	53.5 (21.7)	40.5 (19.7)	−24.4%	>0.99	54.8 (22.5)	2.4%	0.061
MaxOpenVel-Mean (cm/s)	83.7 (34.2)	64.1 (31.0)	−23.4%	>0.99	84.9 (34.3)	1.4%	0.014 *
MaxOpenVel-CV	0.22 (0.12)	0.29 (0.13)	30.6%	>0.99	0.27 (0.12)	19.7%	>0.99
MaxCloseVel-Mean (cm/s)	87.4 (34.4)	66.7 (32.4)	−23.7%	>0.99	90.1 (36.0)	3.1%	0.15
MaxCloseVel-CV	0.23 (0.12)	0.30 (0.13)	32.8%	>0.99	0.27 (0.12)	19.0%	>0.99

* *p* < 0.05 and ** *p* < 0.01 for equivalence assessment with Traditional-MC.

**Table 3:** CART-MMC is sensitive to finger-tapping impairments in PwPD.

Outcome	HC Mean (SD)	PD Mean (SD)	Hedges’ g	*p*-Value
TapCount	46.4 (12.2)	46.3 (12.5)	−0.01	0.96
Freq-Mean (Hz)	3.38 (0.84)	3.42 (0.87)	0.05	0.84
Freq-CV	0.12 (0.06)	0.16 (0.09)	0.57	0.028 *
Amp-Mean (cm)	11.3 (4.1)	8.40 (4.7)	−0.65	0.012 *
Amp-CV	0.24 (0.09)	0.34 (0.15)	0.77	0.005 **
NPL (cm/s)	69.3 (17.9)	51.2 (24.0)	−0.84	0.002 **
MaxOpenVel-Mean (cm/s)	106.2 (27.7)	79.3 (37.2)	−0.81	0.002 **
MaxOpenVel-CV	0.21 (0.07)	0.30 (0.12)	0.91	0.001 **
MaxCloseVel-Mean (cm/s)	112.5 (30.5)	84.9 (39.1)	−0.78	0.003 **
MaxCloseVel-CV	0.21 (0.07)	0.31 (0.13)	0.92	0.001 **

* *p* < 0.05 and ** *p* < 0.01 for difference between groups.

## Data Availability

The data presented in this study are available on request from the corresponding author.

## Abbreviations

The following abbreviations are used in this manuscript:

PD	Parkinson’s disease
PwPD	People with Parkinson’s disease
ADL	Activity of daily living
MDS-UPDRS III	Movement Disorders Society-Unified Parkinson’s Disease Rating Scale Part III
MMC	Markerless motion capture
MC	Motion capture
RGB	Red–green–blue
AR	Augmented reality
HL2	HoloLens2
CART	Comprehensive Augmented Reality Testing
HC	Healthy control
AHaT	Articulated hand tracking
PGM	Portable gray map
LUT	Look-up-table
Dur	Duration
Freq	Frequency
Amp	Amplitude
Vel	Velocity
NPL	Normalized path length
